# The Value of Hepatitis E Screening Sensitivity: Lookback Investigation in German Blood Donors

**DOI:** 10.3390/v18050507

**Published:** 2026-04-28

**Authors:** Ricarda Plümers, Jens Dreier, Attila Mandl, Cornelius Knabbe, Tanja Vollmer

**Affiliations:** 1 Institute for Laboratory and Transfusion medicine, Heart and Diabetes Center North Rhine Westphalia, Ruhr-University Bochum, 32545 Bad Oeynhausen, Germany; 2 Heart and Diabetes Center North Rhine Westphalia, Medical School OWL (Bielefeld University), 32545 Bad Oeynhausen, Germany

**Keywords:** hepatitis E virus, lookback, transfusion-transmitted infections, blood safety

## Abstract

Hepatitis E virus (HEV) is the leading cause of hepatitis globally and poses particular risks for immunocompromised individuals. Mandatory screening of blood donations for HEV RNA and retrospective individual testing of previous donations (lookback investigations) following a reactive result have been implemented in several countries to protect these patients. This includes Germany, where a sensitivity limit of 2000 IU/mL applies to index donations. In total, 334 HEV RNA-positive blood donations were detected at our blood donation service between 2018 and 2024. Lookback testing was applied in 211 cases, revealing previous HEV RNA-positive donations in 23.1% of donors (*n* = 48, 76 donations). Although 16 of these retrospectively tested HEV RNA-positive donations have already been transfused, no transfusion-transmitted HEV infection has been reported. The HEV RNA viral load in the lookback donation was below 50 IU/mL in 72.4% of cases. Routine testing effectively prevents highly viremic blood products entering the supply, significantly reducing the infection risk. While the administration of virus particles with low-viremic products cannot be ruled out, the remaining risk appears to be minimal and has been deemed so far acceptable for the safety of blood products. The lookback strategy further supports the screening strategy by retrospectively identifying blood products from low-viremic donations and enabling appropriate risk management.

## 1. Introduction

The testing of blood donations for hepatitis E virus (HEV) RNA has been mandatory in Germany since 2020 [1]. This requirement is the result of a growing body of knowledge about this infectious disease in recent decades. Starting with studies that revealed seroprevalences between 52.2% and 4.7% in Europe, the awareness and attention regarding HEV have grown [2]. However, the incidences were underestimated for years, as most infections are usually asymptomatic, especially in healthy people [3].

Systematic studies on incidences based on nucleic acid testing (NAT), for example, in blood donors, revealed the high number of unreported cases and the incidences between 1:597 and 1:1447 per year in Germany [4,5,6]. Besides giving insights into the spreading of the virus, this data is interesting for the healthcare system for another reason: HEV infections are a risk factor for non-immunocompetent individuals such as organ donation recipients. An HEV infection is accompanied by an increase in transaminases, jaundice, fatigue and gastrointestinal complaints. It may lead to chronic hepatitis (>6 months) in these patients, which, in its final stage, is associated with cirrhosis of the liver being the final stage of infection [7].

The World Health Organization estimates the number of symptomatic cases at 3.3 million out of 20 million HEV infections per year worldwide [8]. These estimates relate to the infection numbers of all four human pathogenic genotypes. Genotype 3 is predominant in Europe; this is primarily transmitted through the ingestion of undercooked meat, particularly from pigs [9]. In addition, cases of transfusion-transmitted infections (TTIs) with HEV have been described from time to time since 2007 [10,11]. This led to the mandatory testing of blood donations not only in Germany, but also in other European countries such as Austria, France, Ireland, Luxembourg, the Netherlands, Spain and the United Kingdom [12]. The notified body in Germany, the Paul Ehrlich-Institute, has set a minimal detection limit of 2000 IU/mL in a single donation as a cut-off value [1], based on the current limited knowledge regarding TTIs with HEV.

However, HEV is detectable in the blood for only about 4 weeks and in the feces for 6 weeks in asymptomatic individuals and viral loads of HEV in the blood are usually low, peaking at around 2.0 × 10^4^ IU/mL [13]. By comparison, acute hepatitis B virus infections are associated with viral loads of around 10^9^ IU/mL [14]. Moreover, testing in Germany is usually performed in minipools (MPs) of up to 96 samples, leading to a further dilution of the viral load. The interaction of the minimum sensitivity of 2000 IU/mL and low viral doses in asymptomatically infected blood donors might lead to a diagnostic gap. Therefore, not only routine testing of blood donations is required, but also retrospective single analyses of previous donations in case of an HEV RNA-positive donation, as specified by the National Advisory Committee Blood of the Federal Ministry of Health in votum 48 [15].

We summarized the tracing results of HEV RNA-positive donors to gain an insight into how often current testing strategies lead to the release of HEV infected blood products from low viremic donations.

## 2. Materials and Methods

### 2.1. Blood Donors and HEV Nucleic Acid Testing Strategy

The present study includes donations collected at the blood donation service Uni.Blutspendedienst OWL within six years (June 2018 to May 2024). This donation service provides whole blood, platelet, and plasma donations for transfusion purposes as well as plasma for fractionation. The eligibility period for repeat donations varied depending on the type of donation: plasma donations after a minimum of three days, platelet donations after two weeks, and whole blood donations after three to four months. However, donors’ actual donation patterns were often irregular, and they contributed to several types of donation.

In total, 506,943 donations were screened for HEV RNA in MPs of 96 during this period. The RNA/DNA extraction from a 500 µL plasma sample was performed automatically using either the NucliSens easyMAG (bioMérieux, Nürtingen, Germany), the AltoStar AM16 (Altona Diagnostic Technologies, Hamburg, Germany) or the cobas-6000 system (Roche Diagnostics, Basel, Switzerland).

NAT for screening in MP, pool resolution and qualitative testing of lookback donation was time-dependently performed on three different platforms: (1) June 2018 to November 2020: RealStar HEV RT-PCR Kit (Altona Diagnostic Technologies), with a 95% limit of detection (LOD) of 447.36 IU/mL (95% confidence interval (CI): 345.6–720.0 IU/mL) on the Rotor-Gene 3000 system (Corbett Life Sciences, Sydney, Australia); (2) June 2018 to November 2020: AltoStar HEV RNA RT-PCR Kit (Altona Diagnostic Technologies) with a 95% LOD of 327.36 IU/mL (95% CI: 218.88–614.4 IU/mL) on the Bio-Rad CFX96 system (Bio-Rad, Hercules, CA, USA); and (3) November 2020 to May 2024: cobas HEV assay (Roche Diagnostics) with a 95% LOD of 1785.6 IU/mL (95% CI: 1526.4–2169.6 IU/mL) on the cobas-6000 system (Roche Diagnostics). The reported LOD refers to the practical sensitivity in MP of 96. A positive MP containing 96 donations was subdivided into pools of 9 times 10 donations and the remaining 6 donations. The donations contained in the positive sub pool were tested individually. A total of 334 HEV RNA-positive donations have been identified during this period. The donors underwent pre-donation medical evaluations and stated that they were healthy and had no knowledge of an active viral infection. Infections were confirmed by seroconversion using an anti-HEV immunoglobulin G ELISA in a follow-up donation (Euroimmun Labordiagnostika, Lübeck, Germany).

### 2.2. Lookback Procedure

When preparing pools, two aliquots each with 1 mL plasma fraction were set aside as reserve samples from each donation and frozen at −20 °C. The samples are stored for at least one year beyond the shelf life of the preparations. Individual testing and viral load quantification in index donations and positive lookback donations were performed using the AltoStar HEV RNA RT-PCR Kit.

The HEV RNA-positive donations identified by the resolution of the pools (index donations) were not released and disposed. Whether the donor had made a donation that fell under the lookback regulation was checked. The donation with the shortest time interval before the index donation was screened for HEV RNA in an individual test. If this donation took place more than 6 months before the index donation, no lookback analysis was performed. If the lookback donation was negative, the procedure was terminated. If the lookback donation was positive, the previous donation was additionally screened for HEV RNA in an individual test going back to the HEV RNA-negative donation or all donations for up to 6 months prior to the index donation.

In the case of a positive donation not being detected in a MP NAT but in an individual retrospective testing, the blood products needed to be backtracked. Transfusing facilities were informed and surveillance analytics were recommended.

This procedure has been in place at our laboratory since June 2018 and mandatory since November 2020. The lookback strategy is defined by the National Advisory Committee Blood of the German Federal Ministry of Health in votum 48 [15]. It regulates the mandatory testing of whole blood, platelet donations and fresh frozen plasma (FFP). For reasons of complete traceability within the scope of the lookback study, this study also includes retrospectively tested blood plasma donations for fractionation.

The graphical visualization was created with the software GraphPad Prism 9.0 (GraphPad Software, San Diego, CA, USA).

## 3. Results

**Figure 1:**
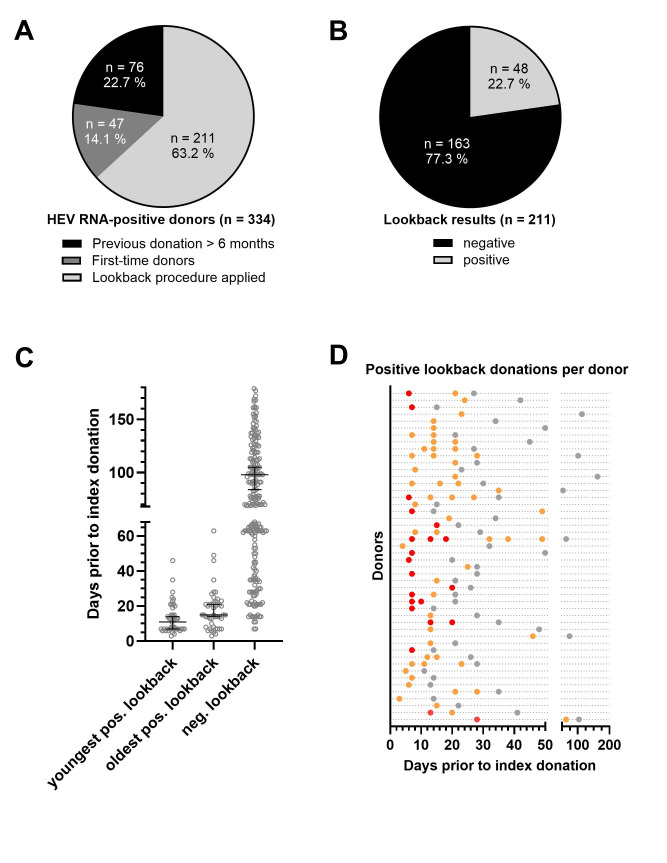
Data summary for lookback procedures. (**A**) Blood donor cohort with HEV RNA-positive index donation for which a lookback procedure has been initiated or not (donation > 6 months prior to the index donation and first donation). (**B**) Proportion of donors with at least one HEV RNA-positive or exclusively negative lookback donations. (**C**) Days between the positive index donation and the youngest positive, oldest positive, and negative lookback donation. Median with 95% CI is given. (**D**) Individual time intervals between the index donation and the HEV RNA-positive donations for each donor with at least one positive lookback (each dotted horizonal line represents one donor). Red dots represent donations with a viral load >50 IU/mL, orange dots represent donations < 50 IU/mL, and gray dots represent negative donations.

A total of 334 blood donors made a donation which was tested positive for HEV RNA (index donation) detectable in MPs during the period under consideration. Data is visualized in Figure 1 for better understanding.

The previous donation was more than 6 months before the index donation in 76 cases; therefore, no lookback investigation was initiated. A total of 47 HEV RNA-positive donors were first-time donors; thus, the procedure did not apply. Consequently, lookback donations from 211 donors were tested for HEV RNA in single tests (Figure 1A). Negative results were found for 163 (76.9%) of them, while 48 donors had at least one or more positive lookback donations (23.1%) (Figure 1B).

Regarding the negative results, the testing was conducted on donations in a median of 91 days (mean 112 days, IQR 64–128 days) before the index donation. Primary positive lookbacks were detected in a median of 11 days (mean 13 days, interquartile range 7–15 days) before the index donation (Figure 1C). The shortest period between a positive index donation and a negative lookback donation was 7 days. The HEV positivity rate was 100% (7 out of 7) among those with an initial lookback donation less than 7 days before the index donation. The HEV positivity rate was 87.5% (14 out of 16) exactly 7 days before index donation. Primary lookback donations made 8 days or more before the index donation were HEV RNA-positive in only 14.4% of cases (27 out of 188).

The longest period between an index and a positive lookback donation was 63 days. Twelve donors had a second HEV RNA-positive lookback donation, four more had three positive lookback donations, one donor had four and one had six HEV RNA-positive retrospective donations. A total of 76 HEV RNA-positive lookback donations were identified over the period under review (Figure 1D).

A total of 76 retrospectively identified HEV RNA-positive donations in the single test HEV-positive lookback donations were identified retrospectively derived from six whole blood donations, ten platelet apheresis procedures, and 60 plasma apheresis procedures.

Product from 23 of these donations was supplied as plasma for fractionation; the purchasers were informed. Blood products from 37 donations were still available and subsequently discarded. Sixteen donations were processed, with 31 resulting blood products being released and distributed. These included four red blood cell concentrates (RBCs), four pooled platelet concentrates (pooled buffy coats from 4 donors), twenty platelet concentrates (PCs) from apheresis and three FFPs. There was no notification of any TTIs to the extent that we have received feedback regarding recipients from the transfusion centers.

Quantification of the viral load revealed values below 50 IU/mL in 55 of the 76 individual tested lookback donations. The remaining 21 had a median viral load of 127.0 IU/mL (mean 180.6 IU/mL; interquartile range 78.0 211.0 IU/mL; maximum 394.0 IU/mL). All of these viral loads were below the detection limit of the initial screening assay regarding the calculated LODs, according to pool size of 96.

A total of 200 HEV RNA-positive donors were identified using the RealStar HEV RT-PCR or the AltoStar HEV RT-PCR Kits (Altona Diagnostic Technologies), respectively, between 2018 and November 2020. Thirty of them had a positive lookback donation (15.0%); ten of those (5.0%) had multiple positive lookbacks. A total of 134 HEV RNA-positive donors were identified using the cobas HEV assay on the cobas6000 (Roche Diagnostics) in the period between November 2020 and June 2024. Eighteen of them (13.4%) had a positive lookback donation; eight of those (5.9%) had multiple positive lookbacks. As there were only four HEV RNA-positive donations with a viral load above 50 IU/mL recognized by the cobas6000 System, a comparison of sensitivities between the two systems based on viral load has been omitted at this point.

## 4. Discussion

The lookback procedure as described by the National Advisory Committee Blood of the German Federal Ministry of Health in votum 48 was applied to 208 out of 334 HEV RNA-positive donations made between June 2018 and May 2024 at our donation service. Forty-eight of them were associated with one or more HEV RNA-positive pre-donations. The median time difference between the primary lookback donation and the index donation was 11 days (mean 13 days). The longest period between a positive lookback and the index donation was 63 days. Acute, self-limiting infections of this duration are uncommon but do occur occasionally, as described elsewhere [13]. Sixteen out of seventy-six HEV RNA-positive blood products were released with no notification of a TTI.

The following discussion will focus on German regulations. The HEV testing of blood donations was introduced in Germany in 2020 with the aim of increasing the safety of blood products and minimizing the risk of infection in immunocompromised patients. Information on minimum infectious doses, technical conditions and the practicability of MP NAT testing led the Paul Ehrlich Institute to set a lower detection limit of 2000 IU/mL in a single donation [1]. The required detection limit was set to protect against the transmission of HEV infection and the development of chronic HEV infection in transplant recipients based on the goal of an 80% risk reduction when testing in 96 MP. Nevertheless, this strategy may not detect all HEV RNA-positive donations due to the presence of low-viremic, asymptomatic donors and the dilution factor resulting from MP NAT.

The infectious dose must be considered to assess the extent to which donors with a viral load below 2000 IU/mL represent a residual risk. The lowest infectious dose described so far leading to the infection of a patient after transfusion was 7 × 10^3^ IU [16,17]. But the lowest infectious dose harbors two disruptive factors that must be well thought-out.

Firstly, the product in question above was a platelet concentrate. However, the type of blood product determines the residual plasma content, which in turn defines the number of units transmitted.

Dynamic processes need to be considered as a second point influencing the evaluation of the infectious dose. While it is assumed that a dose between 7 × 10^3^ and 5 × 10^4^ IU might result in infection with rising probability, the administration of 5 × 10^4^ IU and more is very likely to cause an infection [18]. It is largely accepted that doses above 2 × 10^4^ IU result in efficient transmission (infection in more than 50% of cases), which is determined as the infectious dose for the following considerations.

An estimate of the amount of virus transmitted during a transfusion depending on the blood product and its plasma content can be found in Table 1.

It can be observed at first glance that virus quantities in critical doses might be transfused even with low virus loads if the transfused blood product contains large plasma volumes. The infectious dose is not exceeded in any of the products with the viral load of 50 IU/mL in the donation, with most (55 of 76) of the HEV RNA-positive lookback donations identified being below this value. Nevertheless, when the maximum viral load lookback donation (394 IU/mL) is considered, our data shows that viral loads fall below the infectious dose threshold only in the case of RBC. This emphasizes the importance of the product type when evaluating risk.

In addition, RBCs are derived from whole blood donations. This type of donation poses yet another risk in terms of potentially overlooking donors with low viral loads. Whole blood donations are made at intervals of several months, but healthy donors only experience viremia for a few weeks, therefore, the probability of a whole blood donor making two donations during this phase is very low. While plasma or platelet donations made close together in time can retrospectively identify a low-viremic donation, negatively tested in a MP, the risk of completely overlooking low-viremic donations is higher with whole blood donations.

Although HEV RNA-positive FFPs and PCs pose a risk to patients, it should be pointed out that a large proportion of the positive lookbacks identified do not enter circulation. In particular, the lookback plasma donations made shortly before the index donation (<7 days), which are more likely to be positive and bear a greater risk because of higher viral loads, are often not yet in circulation. The FFPs are often stored and, in the event of a positive single test, discarded in the lookback procedure. The situation is different with PCs. These contain a comparatively high proportion of plasma and are often transfused promptly due to their short shelf life, meaning that they cannot be discarded in the event of a positive lookback test. In our case, this is reflected in the fact that most of the products already distributed were PCs (20 out of 31).

The time frame in which a PC without additive solution, the product with a residual plasma content of 235 mL, would contain an infectious dose will be calculated on a model based on the following. In order to exceed the infectious dose of 2 × 10^4^, the viral load would have to be greater than 85 IU/mL. Here, 7.9 doubling and halving circles are necessary to reach average maximum viral load of 2 × 10^4^ IU/mL and fall below the infectious dose again. Therefore, when considering a half-life or doubling time of 1.6 or 2.4 days, respectively, as published before [13], 13 days before und 19 days after maximal viral load is reached, the viral load would result in a transfusion of 2 × 10^4^ IU and more via a PC. The discussion should take into account the period between reaching the infectious dose of 85 IU/mL and the limit required by the German authorities (2 × 10^3^ IU/mL), which corresponds to 4.6 doubling cycles and a time of 7 days. These calculations are also reflected in the data presented here: 91.3% of donations in the seven days prior to the index donation are positive.

The use of an additive as an alternative to plasma has led to a halving of the plasma content in PCs, which minimizes critical time periods in detection and thus further reduces the risk of TTI with HEV.

Regarding these points, it seems essential to discuss test sensitivity. A residual risk from low-viremia products cannot be ruled out, but is accepted in light of a cost–benefit analysis. A Dutch study found that pool testing (24 MP NAT) significantly reduces the risk of infection, and the cost of testing blood donations per year is one-tenth of the potential treatment costs per patient with chronic HEV infection [19]. Further risk minimization would only be possible through individual testing, which in turn would exceed the treatment costs for chronic TT-HEV. The testing strategy with a detection limit of 2000 IU/mL therefore offers a balance between an 80% risk reduction for patients and simultaneous financial affordability for the healthcare system.

We observed a decline in the number of infections among our blood donors between 2020 and 2022 (cobas-6000 system) compared to the years 2015–2020 (AltoStar system) in a previous study [6]. A connection with the change in the test system could not be ruled out at that time. The concern was that more low-viremic donations had been overlooked due to the lower sensitivity of the last assay used (95% LOD–RealStar Assay: 4.66 IU/mL, AltoStar Assay: 3.41 IU/mL; cobas Assay: 18.6 IU/mL). Consequently, when analyzing the lookback data, we differentiated between the rates from 2018 to 2020 and 2020 to 2024. However, the rates of non-detected low-viremic lookback donations are similar for both periods, with 15% and 13.4%, respectively. Even if it is not possible to evaluate all undetected infections but only those that were identified in following donations, it cannot be assumed that the change of test system had a serious influence on the detection rate.

Various limitations must be taken into account when interpreting our results. No claim to completeness can be made, for example, when it comes to the assessment of overlooked infections. In particular, due to the very short viremia, it is quite possible that a donor never appears at the time of detectable viremia in the MP NAT, no lookback procedure is initiated and a low-viremia donation is not identified. Statistical considerations were also made regarding the transmissibility of an infection with low viral loads in the donations. However, the transmission of infection must always be considered individually, depends on the donor and recipient, and must be interpreted as a probability gradient. Due to inconsistent feedback regarding the post-transfusion monitoring of recipients of HEV RNA-positive blood products, it is not possible to make a definitive statement about the actual risk based on the currently available data. Therefore, our data gives an insight into viral dynamics and the risk of underestimation but is not sufficient to capture the entire risk of undetected infections.

## 5. Conclusions

Sensitivities of available tests and MP NAT strategies have led to the introduction of mandatory HEV screening in blood donors with a limit value of 2000 IU/mL in Germany. The detection borders are smooth, leading to a detection of infections below the limit in some cases. Nevertheless, as our lookback data proves, low-viremic HEV RNA-positive donations could remain undetected, especially within a time window of several days around the index donation. Donors infected with HEV donating only at the edge areas of the diagnostic window might stay undetected anyway. Therefore, only single donation testing would be sufficient to evaluate the actual number of infections among donors. This approach would be time consuming and costly, delivering more information but, as high-viremic products pose a considerably greater risk, with only a few improvements on the safety of blood products. The strategy of lookback testing adds value in the removal of low viremic blood products from the supply system without overtaxing the laboratory conditions.

## Figures and Tables

**Table 1:** Exemplary calculations of the amount of virus transmitted depending on the blood product, which differ in plasma content and transfused volume.

Blood Product	Plasma Content	Transfusion Volume	Transfused Plasma Volume ^1^	Transfused Virus Units	
				Lowest quantifiable viral load (50 IU/mL)	Max. viral load lookback (394 IU/mL)
RBC	5–10%	220–400 mL	40 mL	2.0 × 10^3^ IU	1.5 × 10^4^ IU
PC (apher.)	76—84%	180—280 mL	235 mL	1.2 × 10^4^ IU	9.3 × 10^4^ IU
PC (apher.) +	28—40%	180—280 mL	110 mL	5.5 × 10^3^ IU	4.3 × 10^4^ IU
Pool PC	74—83%	260—360 mL (90—120 mL ^2^)	100 mL	5.0 × 10^3^ IU	3.9 × 10^4^ IU
Pool PC +	40—52%	260—360 mL (90—120 mL ^2^)	60 mL	3.0 × 10^3^ IU	2.4 × 10^4^ IU
FFP	75—82%	200—380 mL	310 mL	1.6 × 10^4^ IU	1.2 × 10^5^ IU
FFP (apher.)	75—91%	190—230 mL	210 mL	1.1 × 10^4^ IU	8.2 × 10^4^ IU

RBC = red blood cell concentrate, PC = platelet concentrate, FFP = fresh frozen plasma, Pool = pooled thrombocytes from three donors, apher. = apheresis, + = in additive, ^1^ calculated from the maximum plasma content and the maximum transfusion volume, ^2^ in relation to the individual donor.

## Data Availability

The original raw data and materials presented in this study will be made available upon request. Further inquiries can be directed to the corresponding author.

## Abbreviations

The following abbreviations are used in this manuscript:

HEV	Hepatitis E Virus
FFP	Fresh frozen plasma
MP	Minipool
NAT	Nucleic acid testing
PC	Platelet concentrate
RBC	Red blood cell concentrate
TTI	Transfusion-transmitted infection
LOD	Limit of detection
